# Elevated CO_2_ ameliorates the high temperature stress effects on physio-biochemical, growth, yield traits of maize hybrids

**DOI:** 10.1038/s41598-024-53343-2

**Published:** 2024-02-05

**Authors:** M. Vanaja, B. Sarkar, P. Sathish, N. Jyothi Lakshmi, S. K. Yadav, Ch. Mohan, A. Sushma, B. S. Yashavanth, M. Srinivasa Rao, M. Prabhakar, V. K. Singh

**Affiliations:** 1 ICAR-Central Research Institute for Dryland Agriculture, Santoshnagar, Hyderabad, TS 500 059 India; 2 ICAR-National Academy of Agricultural Research Management, Rajendranagar, Hyderabad, India

**Keywords:** Physiology, Plant sciences, Climate sciences

## Abstract

The rising temperatures and levels of carbon dioxide in the atmosphere are anticipated to have a significant impact on the productivity of agricultural crops. Although, the individual effects of elevated CO_2_ and temperature have been extensively studied in C3 and C4 crops, there remains a scarcity of research investigating their interactive effects specifically on maize hybrids. The impact of elevated temperature and its interaction with elevated CO_2_ on phenology, physiology, biomass, and grain yield of maize hybrids was assessed in a field experiment using Free Air Temperature Elevation (FATE) facility. The results showed that elevated temperature (eT) increased the anthesis silking interval (ASI), while the presence of elevated CO_2_ along with elevated temperature (eT + eCO_2_) mitigated this effect. The differential expression were observed between hybrids depending on their genetic potential. Furthermore, the net photosynthetic rate (A_net_), stomatal conductance (g_s_), and transpiration rate (Tr) of hybrids decreased under elevated temperature but eT + eCO_2_ condition helped in reverting its impact to some extent. In term of leaf composition, the highest level of total soluble sugars (TSS) and starch was observed under eT + eCO_2_ conditions, possibly due to improved A_net_ in the presence of elevated eCO_2_. The negative impact of eT was also evident through increased proline and MDA content, but eT + eCO_2_ ameliorated the adverse effect of eT. The biomass and grain yield also responded similarly, among the hybrids 900M GOLD recorded superior performance for grain yield at eT condition exceeding 35 °C. On the other hand, DHM117 experienced a significant reduction in grain yield under eT, but performed better under eT + eCO_2_ due to its improved physiological response to eCO_2_. The study indicated that elevated levels of carbon dioxide can actually mitigate the detrimental effects of elevated temperature on maize crop. This positive impact on maize crop can be attributed to an enhanced physiological performance in the presence of eCO_2_ which enables the plants to maintain satisfactory yield levels despite the challenging environmental conditions.

## Introduction

The atmospheric CO_2_ concentration has been increasing at an alarming rate, since the onset of industrial revolution. It was approximately 280 parts per million (ppm) during the pre-industrial period, has now reached around ~ 410 ppm, and it is projected to continue rising in the future^1–3^. Based on the current emission rates, it is projected that CO_2_ levels might increase to 550 ppm by 2050^2^. Climate models indicate that Earth’s near-surface temperature may also experience a significant rise of 1.4 to 5.8 °C as a result of higher concentrations of CO_2_ and other greenhouse gases^4–6^. These predictions underline the urgency of addressing this issue and taking measures to mitigate the impact of climate change.

The elevated atmospheric CO_2_ level is generally known to have a positive effect on net photosynthesis rate, growth, and yield of crops^7–9^. Research has shown that an increase in atmospheric CO_2_ can benefit plant biomass in cereals like barley, wheat, rice, oat, and rye by enhancing the net photosynthesis rate^10–12^. The free-air carbon dioxide enrichment (FACE) studies at eCO_2_ levels also revealed increased photosynthetic rate by almost 40% in different plant species^13,14^. A comprehensive analysis of 186 independent studies conducted on eighteen C3 crops revealed that an elevation of CO_2_ levels led to an average increase in yield by 18%^15^ under non-stress conditions. These findings provide strong evidence for the positive impact of eCO_2_ on crop productivity, indicating that higher concentrations of CO_2_ in the atmosphere can enhance the growth and yield of these crops. The positive effect of elevated CO_2_ (eCO_2_) is more evident in C3 crops compared to C4 crops such as maize^16^. The C4 plants, with their efficient CO_2_ fixation process and carbon concentrating mechanism in bundle sheath cells^17,18^, generally show less stimulation of photosynthesis and growth under eCO_2_ compared to C3 plants. This efficient mechanism allows C4 plants to minimize stomatal conductance, leading to greater drought tolerance compared to C3 species^19,20^. The increase in biomass under eCO_2_ can be attributed to enhanced carbon fixation and an extended active growth^21^ and grain filling period leading to higher crop yields. Additional evidence demonstrating the benefits of eCO_2_, in terms of increased photosynthetic rates, foliar C/N ratio, enhanced plant growth and yield which has also been reported in potato, lettuce, sunflower, barley, and wheat^22–26^.

The increasing CO_2_ levels are also the reason for an increase in ambient temperatures, which influences the crop phenology and duration. The elevated temperature (eT) leads to shorter crop life cycle by reducing the duration of different phenophases in rice, wheat, maize and mung bean^27–29^. The prolonged exposure to high temperatures has been reported to increase photo-respiration in plants^30^ and, also affect the photosystem II activity, chlorophyll concentration, and enzyme activities^31^. The elevated temperature also negatively influences the photosynthesis in addition to heat injury, and metabolic disorders resulting reduced yield^32^. The maize yield in Corn Belt region of US was predicted to reduce by 8% with every 2 °C increase in temperature^33^. In case of wheat production in India, temperature rise of 0.5–1.5 °C decrease the yield potential from 2 to 5%^34^, while an increase by 1 °C is projected to reduce overall production by 4 to 5 million tonnes^35^. An increase in mean canopy temperature by 2.7 °C throughout the crop growing season decreased seed yield in maize, regardless of ambient or elevated CO_2_^36^. In a rice FACE system, elevated CO_2_ increased grain yield by 14%, but there was no significant temperature effect at the relatively cool site^37^. The optimal temperature range for the growth and productivity of maize crops is typically between 22 and 32 °C, with a minimum range of 16.7–23.3 °C. However, extreme temperatures can significantly impact maize productivity. When temperatures drop below 5 °C or rises above 32 °C, it can have adverse effects on the yield of maize crop^38^. Therefore, maintaining temperatures within this suitable range is crucial for maximizing maize productivity. The temperature higher than 32 °C during anthesis silking interval (ASI) in maize drastically affects seed setting. Elevated atmospheric CO_2_ levels can mitigate the adverse effects of moisture deficit stress on maize by reducing stomatal conductance and water loss, while simultaneously preserving soil moisture and maintaining optimal yield^39^.

The studies reveal that eCO_2_ concentration increases the productivity of C3 (15–41%) and C4 (5–10%)^40^. The response of maize crop to elevated CO_2_ indicated a stimulation of biomass by 3–6%^41^, with some reports suggesting an increase of even up to 50%^42^. Elevated CO_2_ enhances maize plant height, leaf area and above ground biomass, resulting in an improved yield and yield related components^43^. The experiments with OTCs showed that eCO_2_ at 550 ppm increased the biomass and grain yield of maize crop^44^. Elevated CO_2_ along with elevated temperature increased stover yield, grain yield and harvest index (HI) of maize compared to ambient CO_2_. The elevated CO_2_ ameliorated the negative impacts of elevated temperature on yield and yield components of maize^28^. These studies revealed that C4 maize crop have the potential to respond positively to elevated CO_2_ like C3 crops. Majority of the studies reported on individual effects of elevated carbon dioxide and temperature on phenology, physiology and biochemistry of different crops^45–49^. Although the combined and interactive effects of elevated CO_2_ and temperature on physiology, phytochemistry, and biomass have been attempted in limited manner^36,50–52^. There is very few research studies to investigate these interactive effects on C4 crops, specifically with maize and also genotypic variability. It is important to understand the interaction of these climate variables on the growth and yield of crop plants, as changes in CO_2_ concentration and temperature are likely to occur concurrently^53^. With this background, the present study was aimed to assess the responses of popular maize hybrids under elevated temperature (eT) and its interaction with elevated CO_2_ (eT + eCO_2_) conditions, focusing on phenology, physiology, biochemical, biomass and grain yield traits.

## Materials and methods

### Plant material and growth conditions

A field study was conducted under Free Air Temperature Elevation (FATE) facility during rainy season for two years, at Central Research Institute for Dryland Agriculture (ICAR-CRIDA) Hyderabad, Telangana, India. The geographical coordinates of the research site are approximately 17.20° N latitude and 78.30° E longitude. The study was to assess the growth and yield responses of four popular maize hybrids (DHM117, DHM121, NK6240, and 900M GOLD) at elevated levels of atmospheric carbon dioxide (eCO_2_) and increased temperature (eT). Among the selected maize hybrids, the 900M GOLD hybrid, developed by Bayer Crop Sciences, stands out for its high-yielding potential. This particular hybrid is well-suited for different regions in India, making it a favourable choice for cultivation across states. Another widely cultivated hybrid is the NK6240, developed by Syngenta Pvt. Ltd. This hybrid has gained recognition for its exceptional adaptability, consistently delivering stable yields in diverse environmental conditions. Additionally, the study included two locally developed hybrids, DHM117 and DHM121 from Telangana State Agricultural University. These hybrids were specifically bred to thrive in the rainfed conditions prevalent in Telangana State.

The FATE facility consisted of nine rings, each with a diameter of 8 m, enabling the maintenance of three treatment conditions. This design allowed for controlled experimentation and the examination of the effects of elevated temperature and CO_2_ on maize hybrids within the specific planting configuration. The crop was sown with a spacing of 0.30 m between plants and 0.75 m between rows. It's worth noting that the plant density within the FATE rings was intentionally set different from the recommended spacing of 0.25 m (plant to plant) and 0.60 m (row to row), typically followed in rainfed agro-ecologies. This specific spacing was chosen to ensure that each plant is exposed to elevated levels of temperature and CO_2_ released by the infrared heater and CO_2_ system within the ring.

**Plate 1:**
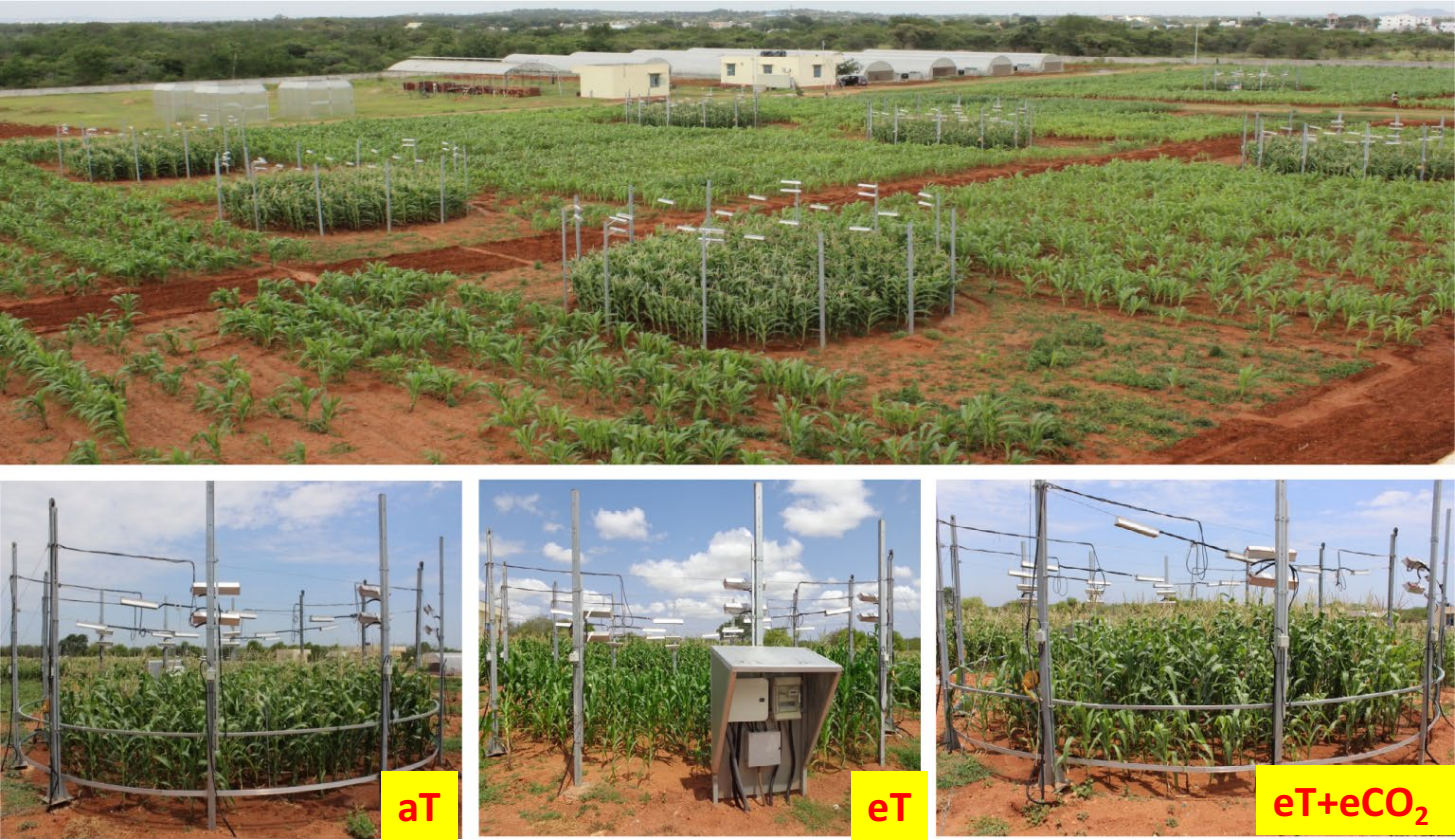
Experiment under FATE facility.

The study consisted of three distinct treatment levels. The first level served as the control treatment, maintaining ambient conditions with a CO_2_ concentration of ~ 400 ppm and the temperature at ambient levels (aT). The second level involved an elevated temperature (eT), with the temperature set at ambient levels + 3 °C ± 0.5 °C. The third treatment level combined elevated temperature and CO_2_ (eT + eCO_2_), simulating environmental conditions with both the temperature set at ambient levels + 3 °C ± 0.5 °C and elevated CO_2_ level of 550 ± 50 ppm. Three FATE rings having similar treatment conditions were used as three replications (Plate 1). Each ring, with a total area of 50.26 m^2^, was further divided into four equal quadrants, with each quadrant having an area of 12.56 m^2^. This division was done to allocate space for sowing each hybrid in each quadrant, ensuring a randomized distribution of the hybrids across the different rings. By implementing this design, the study aimed to minimize any potential bias that could arise from the placement of the hybrids within the experimental setup.

To maintain elevated crop canopy temperature (eT) of ambient + 3 °C ± 0.5 °C, each warming ring was fitted with 24 ceramic infrared heaters assembly consisting each of 2 × 1000 W capacity (Elstein, model FSR-1000W) above the canopy. The heating system provides warming without any photo-morphogenic effects or significant radiation emitted below 850 nm wavelengths. Out of six warming rings, three rings were also provided with CO_2_ release system at 0.30 and 0.8 m height from the base of the ring to assess the interactive effects of elevated temperature and CO_2_. The polyurethane (PU) tubing along the fringe of the ring with laser drilled perforations releases CO_2_ within ring to maintain the elevated concentration of 550 ppm. The CO_2_ release was controlled by solenoid valves, which in turn was regulated by the SCADA based control system linked with CO_2_ analyser, wind direction and wind speed. The CO_2_ concentration at the centre of the ring was continuously monitored by IRGA based CO_2_ analyser (Priva, model-200821) and the duration of CO_2_ release was based on the set CO_2_ concentration for the specified area as well as wind direction and wind speed. The canopy temperatures were monitored with infrared sensor (Ray teck Fluke, model-RAYCMLTJ3) fitted in each ring. The duration and intensity of heating of warming plots is regulated by proportional-integral-derivative (PID) system using canopy temperatures of control plots as reference^11^. The set conditions were maintained for 24 h throughout the crop season, starting from the germination and continued until the physiological maturity of the crop. The signals from each sensor are being monitored, recorded and controlled by Program Logic Control (PLC) and Supervisory Control and Data acquisition (SCADA) system. The recommended doses of fertilizers were applied in three splits @ 60 kg N ha^−1^ and 60 kg P ha^−1^, 30 kg K ha^−1^ as muriate of potash as basal; 30 kg N ha^−1^ as second dose at knee- high stage and 30 kg N ha^−1^ and 30 kg potassium ha^−1^ as third dose was applied at tasselling stage. The crop was raised with supplemental irrigation at regular intervals and maintained at optimum soil moisture along with recommended plant protection measures to control pests and diseases.

### Weather during crop growth period

**Figure 1:**
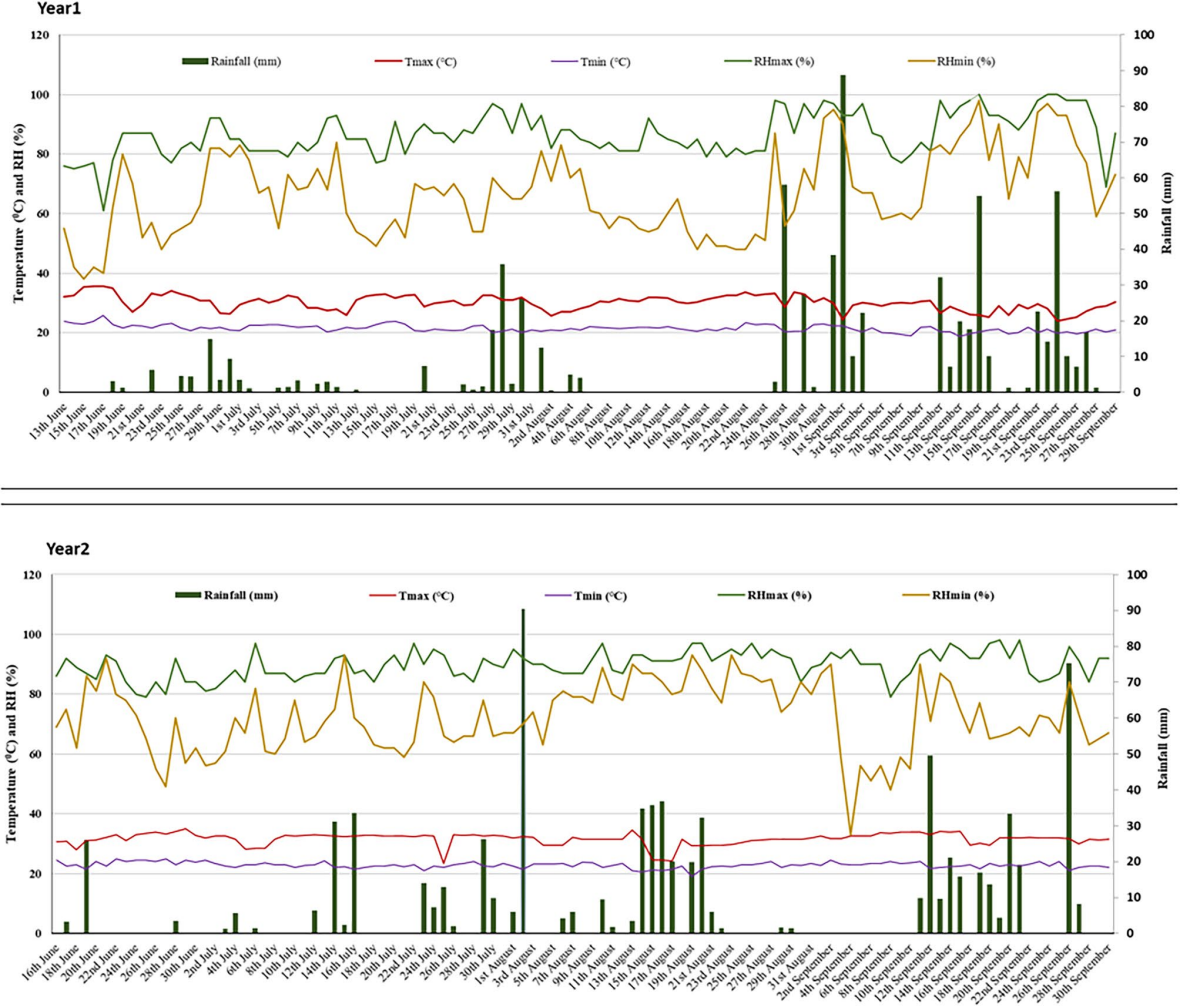
Weather parameters of two crop seasons where the experiments was conducted.

The weather parameters of two seasons during crop growth period were presented in Fig. 1. In season one, the maximum air temperature during vegetative stage of the crop ranged from 25.6 to 35.6 °C with an average value of 30.84 °C while minimum temperature ranged from 20.0 to 25.7 °C with an average value of 21.9 °C. While, during grain filling stage, maximum temperature ranged from 24.0 to 33.6 °C with an average value of 29.47 °C and minimum temperature from 18.8 to 23.4 °C with an average value of 21.1 °C. In season two, during vegetative stage of the crop, the maximum air temperature ranged from 23.4 to 35.0 °C with an average value of 31.9 °C while minimum temperature ranged from 21.0 to 25.0 °C with an average value of 23.1 °C. During grain filling stage, maximum temperature ranged from 24.2 to 34.6 °C with an average value of 31.4 °C and minimum temperature from 19 to 24 °C with an average value of 22.7 °C. The maximum humidity in first season varied between 61 to 100%, while minimum was from 38 to 98%. In the first season crop received 700 mm rainfall. In case of second season, maximum humidity varied between 79 to 98%, while minimum was from 33 to 93% and crop received a total of 770 mm rainfall.

### Observations recorded

#### Phenological parameters

The phenological observations of days to 50% tasselling, anthesis and silking were recorded when 50% of the plants had tassel initiated, pollen shed and had emerged silks respectively. The anthesis silking interval (ASI) was calculated as number of days between days to anthesis and days to silking.

#### Physiological parameters

All physiological parameters were recorded on the fully expanded third leaf from the top. The relative water content (RWC) was determined following standard protocol^54^ i.e. RWC (%) = [(FW − DW)/(TW − DW)] × 100, where FW—fresh leaf weight, TW—turgid leaf weight after rehydration, DW—the dry leaf weight after oven drying.

The net photosynthetic rate (A_net_), stomatal conductance (g_s_), transpiration rate (Tr), internal CO_2_ concentration (Ci), leaf temperature (Tleaf) and vapour pressure deficit (VPD) were measured during anthesis stage. The measurement was taken on fully expanded third leaf from uppermost active leaf in three representative plants for each hybrid. The measurements were recorded between 10:00 and 12:00 h using portable photosynthesis system (LI-6400, LI-COR, Nebraska, USA) with irradiance set at 1200 µmol m^−2^ s^−1^ under three conditions viz., aT, eT, and eT + eCO_2_. The conditions of temperature and CO_2_ in the leaf chamber of IRGA were set as that of the plot conditions. The water use efficiency (WUE) was calculated as the ratio of A_net_ and Tr using the formula WUE = A_net_/Tr.

#### Biochemical parameters

To estimate alcohol soluble metabolites, fresh leaf tissue of 0.5 g was homogenized in 80% ethanol by grinding using mortar and pestle. The homogenized sample was centrifuged at 24 °C, 10,000 rpm for 20 min and the supernatant was stored in screwcap tubes in refrigerator. The supernatant was used for estimation of total soluble sugars, free amino acids and starch content.

Total soluble sugars were estimated by the sulphuric acid-phenol method^55^, where 0.1 mL of supernatant was taken in a test tube and 1 mL of phenol reagent was added followed by 5 mL of concentrated sulphuric acid and mixed thoroughly. The samples were incubated for 30 min at room temperature and after the colour development absorbance was measured at 490 nm by using spectrophotometer and expressed as mg g^−1^ fresh leaf weight.

The free amino acids content was determined by using method of Moore and Stein^56^, where 0.1 mL of supernatant was taken in test tube and mixed with 1 mL of freshly prepared ninhydrin reagent and volume was made up to 2 mL with distilled water and then heated in boiling water bath for 20 min. After cooling, 5 mL of propanol was added to the above mixture and kept for 15 min. The absorbance was read at 570 nm by using spectrophotometer and expressed as mg g^−1^ fresh leaf weight.

Starch content was determined by anthrone method^57^. The residue (pellet) was washed repeatedly with 80% ethanol till the washing does not give colour with anthrone reagent and then dried. Water and perchloric acid (52%) at the ratio of 1:1 was added into pellet and centrifuged. The process was repeated twice and obtained supernatant was used for measurement of starch content. The absorbance was read at 630 nm by using spectrophotometer and expressed as mg g^−1^ fresh leaf weight.

The lipid peroxidation was estimated in terms of malondialdehyde (MDA) content following Health and Packer^58^ method. To estimate MDA content, 1.0 g of leaf tissue was grinded in 2.0 mL of TBA and centrifuged at 10,000 rpm for 10 min at 4 °C. In a test tube two mL of supernatant and 4 mL of 0.5% TBA were added. The test tubes covered with aluminium foil were heated at 95 °C for 1 h and immediately cooled in ice bath. The mixture was centrifuged for 5 min at 10,000 rpm. Then the supernatant was collected and the absorbance was read at 532 and 600 nm by using spectrophotometer to measure the MDA content and expressed as μmol/g FW.

Proline content was estimated following Bates^59^ method. One gram of fresh leaf tissue was homogenized in 3% aqueous sulphosalicylic acid using mortar and pestle and centrifuged at 10,000 rpm at 24 °C for 10 min. Acid-ninhydrin solution (1.25 g ninhydrin in 30 mL glacial acetic acid) was added and heated at 90 °C for 1 h. The reaction was terminated by placing the tubes in ice bath, and then extracted with 4 mL of toluene by vortexing for 1 min. The absorbance was read at 520 nm in spectrophotometer using toluene as a blank and expressed as μM proline/g of fresh leaf weight.

#### Biomass and yield parameters

At the maturity, three plants of each hybrid from every replication under three different conditions (aT, eT and eT + eCO_2_) were carefully uprooted. These plants were then separated into their respective components, including leaves, stems, roots, and cobs. To ensure accurate measurements, the roots were thoroughly washed to remove any soil particles. Subsequently, the harvested plant parts were subjected to a drying process in a hot air oven set at 55 °C. The drying continued until the plant samples reached a constant weight for determination of dry weights. The dry weight of leaf, stem, and root was measured using scientific balance and expressed as gram per plant. The yield parameters—cob weight (g/plant), seed number, seed yield (g/plant), test weight (hundred seed weight), total biomass, vegetative biomass, and harvest index (HI) was recorded. The entire experimental setup was conducted under controlled conditions with predefined parameters and hybrid, being one hundred percent heterozygous and homogeneous in nature, the average of three plants data per replication represent its actual responses to different treatments. Harvest index was calculated as $HI=\left(\frac{Grain\, yield}{Total\, biomass}\right)\times 100$ and expressed in percentage.

### Statistical analysis

The replicated data of individual season and combined over seasons were subjected to statistical analysis following randomized complete block design (RCBD) using SAS software ver. 9.3 to assess the significance of treatments, hybrids and their interaction. The analysis of variance was applied to compare hybrids in individual trial and combined over treatments. Subsequently, the Tukey’s Honest Significant Difference (Tukey’s HSD) test was used post-hoc to identify the significant treatments and hybrids. All statistical tests were conducted at 5% level of significance. The R statistical programming language was used to visualize the results from ANOVA and Tukey’s HSD test.

### Ethics declarations

All plant experiments were conducted in accordance to relevant institutional, national, and international guidelines and legislation.

## Results

The combined analysis of variance (ANOVA) revealed significant variances for most of the traits related to phenological, physiological, biochemical, biomass and yield traits due to hybrids, treatments and treatments × hybrids interaction (Table 1).

**Table 1:** Combined analysis of variance (ANOVA) for physiological, biochemical and yield related parameters.

Source	df	Mean sum of square												
		Anthesis	Silking	ASI	LDW	SDW	RDW	TBM	VBM	CW	SN	HSW	SY	HI
Rep (year)	5	12.36**	8.73**	0.56	235.5**	697.5**	198.8**	3127.6**	1651.06**	44.088	114.18	4.5	246.43**	7.51
Treatments	2	135.5**	92.17**	4.17**	238.1**	1116.39**	3.88	10,033.2**	3300.34**	4002.38**	9594.18**	27.11**	1857.72**	1.95
Hybrids	3	45.09**	43.5**	1.59	396.06**	836.49**	137.13**	1327.8**	1025.52**	811.56**	22,275.67**	57.87**	731.86**	47.36**
Treatments* hybrids	6	0.76	2.56	1.43	17.82	95.75**	17.65*	1030.29**	359.08*	353.65**	1667.24**	10.66**	310.95**	7.15
Error	55	2.051	1.96	0.68	17.49	41.01	7.48	191.6	147.98	112.19	476.86	5.71	58.93	4.62

Source	df	Mean sum of square												
		A_net_	g_s_	Tr	WUE	RWC	MDA	Proline	FAA	TSS	Starch	Tleaf	Ci	VPD
Rep (year)	5	6.94	0.038**	16.37**	20.93**	10.46**	51.10**	208.76**	3.39**	0.083	9.32**	0.37**	13,268.95**	0.26**
Treatments	2	706.45**	0.084**	8.47**	5.72**	507.86**	103.07**	1016.33**	9.97**	50.99**	76.57**	26.57**	107,109.05**	1.14**
Hybrids	3	53.79**	0.004	0.54	0.53*	16.72**	8.48**	5.22	2.23**	11.63**	18.21**	0.134	525.08	0.02*
Treatments* hybrids	6	33.69**	0.017**	3.24**	1.07**	35.25**	8.83**	87.96**	0.33**	1.44**	11.86**	0.29*	3762.78**	0.04**
Error	55	7.14	0.002	0.3	0.164	3.07	0.73	4.18	0.244	0.342	0.801	0.09	488.76	0.005

*df* degrees of freedom, *ASI* anthesis silking intervals, *LDW* leaf dry weight (g/pl.), *SDW* stalk dry weight (g/pl.), *RDW* root dry weight (g/pl.), *TBM* total biomass (g/pl.), *VBM* vegetative biomass (g/pl.), *CW* Cob weight (g/plant), *SN* seed number, *HSW* hundred seed weight (g), *SY* seed yield (g/plant), *HI* harvest index, *A*_*net*_ photosynthetic rate (μmol CO_2_ m^−2^ s^−1^), *g*_*s*_ stomatal conductance (mol m^−2^ s^−1^), *Tr* transpiration rate (mmol H_2_O m^−2^ s^−1^), *WUE* water use efficiency (µmol CO_2_ mmol H_2_O^−1^), *RWC* relative water content (%), *MDA* malondialdehyde content (mg/g F.Wt), proline (μg/g F.Wt), *FAA* free amino acids (mg/g F.Wt), *TSS* total soluble sugars (mg/g F.Wt), starch (mg/g F.Wt), *Tleaf* leaf temperature (°C), *Ci* internal CO_2_ in ppm, *VPD* vapour pressure deficit (K Pa).

* and ** significant at 5% and 1% level of significance respectively.

### Phenology

**Figure 2:**
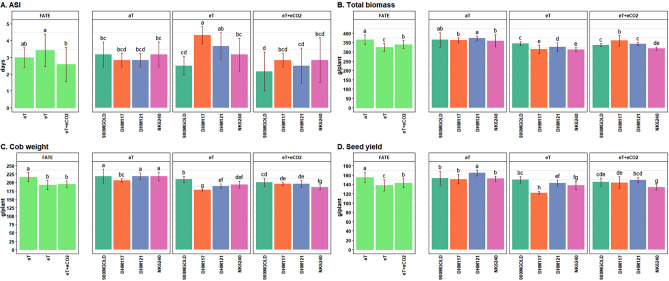
Performance of maize hybrids under different treatment conditions for crop phenology and yield related traits. Data are given as mean ± SD. Treatments with different grouping letters are significantly different.

There was differential responses of anthesis and silking among hybrids under elevated temperature (eT) which influenced the crop phenology, specially affecting the days to anthesis and silking (Table 2) resulting increase in ASI as compared to aT. However, under eT + eCO_2_ conditions, presence of eCO_2_ partially mitigated the negative effects of eT (Fig. 2A). Among hybrids tested, DHM117 had the highest ASI (4.33 days), followed by DHM121 (3.67 days), NK6240 (3.17 days) and 900M GOLD (2.5 days) under eT conditions (Table 3).

**Table 2:** Means of phenology and biomass parameters at different treatment level.

Treatments	Anthesis	Silking	ASI	LDW	SDW	RDW	TBM	VBM
aT	56.33^b^	59.33^b^	3.00^ab^	44.20^a^	84.34^a^	20.04^a^	364.21^a^	209.06^a^
eT	53.83^c^	57.25^c^	3.42^a^	38.20^b^	72.99^b^	20.26^a^	323.81^c^	195.75^b^
eT + eCO_2_	58.58^a^	61.17^a^	2.58^b^	39.54^b^	85.22^a^	19.48^a^	338.53^b^	185.68^c^
GM	56.25	59.25	3.00	40.65	80.85	19.93	342.18	196.83
SEM	0.15	0.15	0.17	0.65	0.98	0.35	1.60	1.43
CV (%)	1.29	1.23	27.76	7.85	5.94	8.67	2.29	6.18
LSD_0.05_	0.42	0.42	0.48	1.85	2.79	1.00	4.55	7.03

*aT* ambient canopy temperature, *eT* elevated canopy temperature, *eT* + *eCO*_*2*_ elevated temperature (eT) and its interaction with elevated CO_2_, *GM* mean of all the hybrids, *SEM* standard error of mean difference between treatments, *CV* coefficient of variation, *LSD*_*0.05*_ least significant differences between treatments at 5% level of significances.

The alphabetical letters given as superscript against each values indicates significant differences between treatments at P ≤ 0.05 if letters are different for different treatments, otherwise non-significant if having same letters.

**Table 3:** Means of phenology and biomass parameters at treatment × hybrids level.

Treatments	Hybrids	Anthesis	Silking	ASI	LDW	SDW	RDW	TBM	VBM
aT	900M GOLD	57.83^b^	61.00^b^	3.17^bc^	49.36^a^	78.68^cd^	17.27^cd^	364.00^ab^	211.34^ab^
	DHM117	57.17^b^	60.00^c^	2.83^bcd^	46.80^ab^	89.29^b^	19.11^c^	361.70^b^	210.63^ab^
	DHM121	56.17^c^	59.00^d^	2.83^bcd^	39.38^de^	88.93^b^	24.77^a^	371.91^a^	207.45^b^
	NK6240	54.17^d^	57.33^f^	3.17^bc^	41.24^cd^	80.48^c^	19.01^c^	359.22^b^	207.13^b^
eT	900M GOLD	54.83^d^	57.33^f^	2.50^cd^	44.47^bc^	73.36^d^	16.93^d^	344.21^c^	194.40^c^
	DHM117	54.50^d^	58.83^de^	4.33^a^	40.39^d^	77.99^cd^	17.75^cd^	313.50^e^	191.62^cd^
	DHM121	54.33^d^	58.00^ef^	3.67^ab^	35.40^f^	76.39^cd^	24.24^a^	325.52^d^	182.56^e^
	NK6240	51.67^e^	54.83^g^	3.17^bc^	32.51^f^	64.23^e^	22.13^b^	312.01^e^	174.15^f^
eT + eCO_2_	900M GOLD	59.50^a^	61.67^b^	2.17^d^	42.25^cd^	76.87^cd^	16.25^d^	335.66^c^	190.74^cd^
	DHM117	59.83^a^	62.67^a^	2.83^bcd^	44.76^bc^	98.06^a^	21.14^b^	359.98^b^	216.31^a^
	DHM121	59.00^a^	61.50^b^	2.50^cd^	35.72^ef^	88.60^b^	21.51^b^	341.43^c^	192.26^c^
	NK6240	56.00^c^	58.83d^e^	2.83^bcd^	35.42^f^	77.34^cd^	19.03^c^	317.04^de^	183.71^de^
SEM		0.3	0.3	0.34	1.3	1.96	0.71	3.2	2.87
LSD_0.05_		0.85	0.85	0.97	3.7	5.58	1.42	9.11	8.17

*SEM* standard error of mean difference between for treatments × hybrid interaction.

The alphabetical letters given as superscript against each values indicates significant differences between treatments at P ≤ 0.05 if letters are different for different treatments, otherwise non-significant if having same letters.

### Biomass

There were significant differences between hybrids under elevated temperature for total biomass, and its vegetative and yield components (Table 2). Among the hybrids, the reduction of leaf biomass at eT varied from 5.5% (900M GOLD) to 15% (NK 6240) and total biomass from 5.44% (900M GOLD) to 13.3% (DHM117). Notably, the hybrid DHM117 exhibited better recovery in leaf, shoot, and total biomass under eT + eCO_2_ condition, reaching levels comparable to the ambient treatment (Table 3). Under ambient condition, the total biomass was highest and similar across all hybrids, followed by eT + eCO_2_ and eT. Additionally, the hybrid 900M GOLD recorded significantly higher biomass under eT as compared to other hybrids, whereas, DHM117 and DHM121 demonstrated the ability to recover under eT + eCO_2_ condition (Fig. 2B).

### Yield parameters

The combined analysis of variance (ANOVA) revealed highly significant (p < 0.01) variances for cob weight, seed number, seed yield, and hundred seed weight due to hybrids, treatments and their interaction. The impact of elevated temperature was found to be significant in reducing the yield components however, the magnitude of response varied with individual hybrid (Table 4). Specifically, NK6240, DHM117, and DHM121 experienced reduction in cob weight by 11.61%, 14.11% and 13.41% respectively (Fig. 2C). The most significant reduction in seed yield (19.32%) was observed with DHM117 (Fig. 2D) which is primarily attributed due to a decrease in seed number (12.67%). Among the four maize hybrids, 900M GOLD had higher cob weight, seed number, and hundred seed weight as it responded positively for yield components under both eT and eT + eCO_2_ conditions (Table 5). It demonstrated the least reduction in cob weight (4.23%), seed yield (2.06%) under eT conditions, also showing an increase in hundred seed weight (5.13%) indicating its tolerance to high temperature.

**Table 4:** Means of grain yield related traits at different treatment levels.

Treatments	Cob weight	SN	Seed Yield	HSW	HI (%)
aT	215.63^a^	498.46^a^	155.15^a^	31.38^a^	42.64^a^
eT	192.36^b^	469.96^b^	138.13^c^	29.44^b^	42.73^a^
eT + eCO_2_	194.29^b^	459.92^c^	142.77^b^	31.17^a^	42.20^a^
GM	200.76	476.11	145.35	30.66	42.52
SEM	1.41	1.52	1.01	0.22	0.28
CV (%)	3.44	1.57	3.41	3.49	3.21
LSD_0.05_	4.01	4.33	2.87	0.62	0.79

The alphabetical letters given as superscript against each values indicates significant differences between treatments at P ≤ 0.05 if letters are different for different treatments, otherwise non-significant if having same letters.

**Table 5:** Means of grain yield parameters at treatment × hybrids level.

Treatments	Hybrids	CW	SN	SY	HSW	HI (%)
aT	900M GOLD	218.69^a^	556.67^a^	152.96^b^	27.68^g^	42.04^cd^
	DHM117	206.50^bc^	498.33c	151.07^b^	30.32^bcd^	41.73^d^
	DHM121	218.83^a^	487.83^d^	164.46^a^	33.71^a^	44.23^a^
	NK6240	218.50^a^	451.00^g^	152.09^b^	33.82^a^	42.55^bcd^
eT	900M GOLD	209.45^b^	515.83^b^	149.81^bc^	29.10^def^	43.52^abc^
	DHM117	177.37^g^	435.17^h^	121.88^h^	28.11^fg^	39.03^e^
	DHM121	189.48^ef^	479.00^e^	142.96^ef^	29.87^cde^	44.15^a^
	NK6240	193.14^def^	449.83^g^	137.86^fg^	30.70^bc^	44.23^a^
eT + eCO_2_	900M GOLD	200.30^cd^	503.00^c^	144.93^cde^	28.89^efg^	43.16a^bcd^
	DHM117	196.02^de^	463.50^f^	143.68^de^	30.98^bc^	39.89^e^
	DHM121	195.61^de^	448.67^g^	149.17^bcd^	33.25^a^	43.69^ab^
	NK6240	185.25^fg^	424.50^i^	133.33^g^	31.56^b^	42.05^cd^
SEM		2.82	3.05	2.02	0.44	0.56
LSD_0.05_		8.03	8.67	4.07	1.24	1.59

*SEM* standard error of mean difference between for treatments × hybrid interaction.

The alphabetical letters given as superscript against each values indicates significant differences between treatments at P ≤ 0.05 if letters are different for different treatments, otherwise non-significant if having same letters.

### Physiological parameters

**Figure 3:**
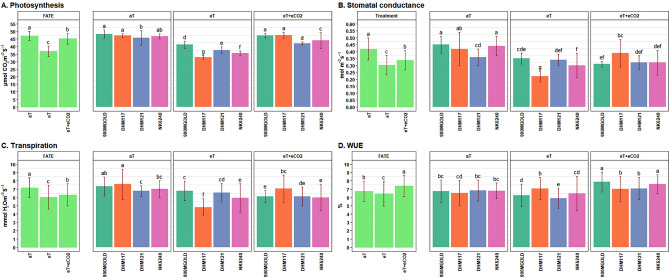
Performance of maize hybrids under different treatment conditions for physiological traits. Data are given as mean ± SD. Treatments with different grouping letters are significantly different.

The ANOVA revealed significant variances attributed to the treatment, treatment × hybrids interaction (p < 0.01) for A_net_, g_s_, Tr, leaf temperature, internal CO_2_ and vapour pressure deficit. The crops grown under eT condition had significantly lower values of A_net_, g_s_ and Tr compared to those under aT condition (Fig. 3A–C). The A_net_ was particularly affected by eT, the crop showed some degree of recovery under eT + eCO_2_ (Table 6). Both transpiration rate and stomatal conductance also decreased significantly under eT. Leaf temperature was significantly lower under aT as compared to eT and eT + eCO_2_ while internal CO_2_ content was lowest under eT followed by aT and eT + eCO_2_. The VPD was significantly higher under eT as compared to aT, followed by eCO_2_ + eT. Among the hybrids, 900M GOLD maintained relatively higher A_net_, g_s_, and Tr at eT, indicating its ability to capture more CO_2_. The A_net_ of all hybrids reduced under eT but, the presence of eCO_2_ facilitated its recovery in DHM117 and 900M GOLD, reaching levels similar to those in the ambient plots. DHM121 and NK6240 showed > 98% recovery under eT + eCO_2_ condition. Among the maize hybrids, 900M GOLD consistently maintained highest A_net_ under all the three conditions, with the lowest impact of eT. Although DHM117 experienced the greatest reduction of A_net_ (28.4%) under eT, it was able to recover to that ambient levels in the presence of eCO_2_ (Table 7). The leaf temperature increased significantly in hybrids under eT and eT + eCO_2_ as comparison to aT. In contrast, internal CO_2_ decreased under eT but showed a significant increase under eT + eCO_2_ conditions. However, the response of genotypes varied, particularly in the case of DHM117, where internal carbon significantly decreased under eT, possibly due to stomatal closure, leading to a reduced A_net_. Conversely, DHM121 exhibited higher internal CO_2_ under eT as compared to aT, aligning with a lesser impact on A_net_ and stomatal conductance (g_s_), and also maintaining better RWC resulting in the higher accumulation of CO_2_. Furthermore, the vapor pressure deficit (VPD) among hybrids was markedly higher under eT compared to aT and showed a reduction in the presence of eCO_2_ under the eT + eCO_2_ conditions.

**Table 6:** Means of physiological and biochemical parameters at different treatment level.

Treatments	A_net_	g_s_	Tr	WUE	RWC	MDA	Proline	FAA	TSS	Starch	Tleaf	Ci	VPD
aT	47.04^a^	0.420^a^	7.17^a^	6.74^b^	87.27^a^	13.95^c^	21.00^c^	6.63^a^	6.06^c^	13.42^b^	29.34^b^	178.88^b^	1.88^c^
eT	36.88^c^	0.302^c^	6.03^c^	6.43^c^	78.12^c^	18.05^a^	33.85^a^	5.66^b^	7.26^b^	13.26^b^	31.20^a^	166.80^b^	2.32^a^
eT + eCO_2_	45.25^b^	0.337^b^	6.32^b^	7.39^a^	83.54^b^	15.49^b^	25.66^b^	5.42^c^	8.96^a^	16.43^a^	31.11^a^	288.08^a^	2.16^b^
GM	43.06	0.353	6.51	6.85	82.98	15.83	26.84	5.9	7.43	14.37	30.55	211.25	1.77
SEM	0.27	0.01	0.08	0.07	0.39	0.16	0.2	0.05	0.08	0.07	0.06	3.25	0.01
CV (%)	3.05	9.76	6.35	4.93	2.32	5.02	3.66	4.18	5.5	2.36	1.02	10.46	3.35
LSD_0.05_	0.77	0.02	0.24	0.2	1.12	0.46	0.57	0.14	0.24	0.2	0.18	12.79	0.04

The alphabetical letters given as superscript against each values indicates significant differences between treatments at P ≤ 0.05 if letters are different for different treatments, otherwise non-significant if having same letters.

**Table 7:** Means of physiological and biochemical parameters at treatment × hybrids level.

Treatments	Hybrids	A_net_	g_s_	Tr	WUE	RWC	MDA	Proline	FAA	TSS	Starch	Tleaf	Ci	VPD
aT	900M GOLD	48.22^a^	0.448^a^	7.32^ab^	6.76^bc^	87.58^a^	14.24^fg^	23.45^fg^	6.78^a^	5.01^g^	13.22^e^	29.56^c^	189.00^cd^	1.81^g^
	DHM117	47.35^a^	0.420^a^b	7.59^a^	6.54^cd^	87.51^a^	14.11^g^	19.91^i^	6.66^a^	6.97^e^	14.25^c^	29.60^c^	178.67^cde^	2.03^e^
	DHM121	45.77^b^	0.359^cd^	6.78^c^	6.85^bc^	87.54^a^	12.78^h^	19.28^i^	6.32^b^	6.09^f^	11.74^g^	29.02^d^	154.38^f^	1.92^f^
	NK6240	46.83^ab^	0.440^a^	7.01^bc^	6.79^bc^	86.44^a^	14.65^efg^	21.36^h^	6.77^a^	6.15^f^	14.49^c^	29.17^d^	193.50^cd^	1.80^g^
eT	900M GOLD	41.25^d^	0.352^cde^	6.78^c^	6.27^d^	82.23^b^	15.99^cd^	31.27^c^	5.54^c^	6.80^e^	13.34^de^	31.26^ab^	178.17^de^	2.31^bc^
	DHM117	32.98^g^	0.223^g^	4.80^f^	7.09^b^	75.32^d^	20.47^a^	40.03^a^	5.72^c^	8.04^cd^	13.32^de^	31.02^b^	130.50^g^	2.25^c^
	DHM121	37.63^e^	0.335^def^	6.59^cd^	5.88^e^	78.45^c^	17.83^b^	31.21^c^	5.23^d^	7.19^e^	12.77^f^	31.12^ab^	196.83^c^	2.33^ab^
	NK6240	35.65^f^	0.297^f^	5.94^e^	6.49^cd^	76.47^cd^	17.90^b^	32.90^b^	6.15^b^	7.03^e^	13.62^d^	31.40^a^	161.73^ef^	2.39^a^
eT + eCO_2_	900M GOLD	47.35^a^	0.313^ef^	6.11^e^	7.87^a^	82.93^b^	15.13d^ef^	24.08^f^	5.53^c^	8.35^c^	15.66^b^	31.01^b^	266.83^b^	2.12^d^
	DHM117	47.55^a^	0.388^bc^	7.06^bc^	7.02^b^	86.65^a^	15.04^ef^	22.76^g^	5.06^d^	10.65^a^	19.79^a^	31.23^ab^	302.00^a^	2.17^d^
	DHM121	42.12^d^	0.319^def^	6.13^de^	7.08^b^	81.29^b^	15.52^cde^	29.73^d^	4.95^d^	8.98^b^	15.73^b^	31.15^ab^	297.33^a^	2.17^d^
	NK6240	44.00^c^	0.324^def^	6.00^e^	7.59^a^	83.27^b^	16.26^c^	26.06^e^	6.12^b^	7.85^d^	14.55^c^	3107^ab^	286.17^a^	2.17^d^
GM		43.06	0.35	6.51	6.85	82.97	15.83	26.84	5.90	7.43	14.37	30.55	211.25	2.12
SEM		0.54	0.01	0.17	0.14	0.79	0.32	0.40	0.10	0.17	0.14	0.12	6.50	0.02
CV (%)		3.05	9.76	6.35	4.93	2.32	5.02	3.66	4.18	5.50	2.36	0.98	7.54	2.43
LSD_0.05_		1.53	0.04	0.48	0.39	2.24	0.92	1.14	0.29	0.48	0.40	0.35	18.51	0.06

Significant values are in bold.

The alphabetical letters given as superscript against each values indicates significant differences between treatments at P ≤ 0.05 if letters are different for different treatments, otherwise non-significant if having same letters.

The reduction in g_s_ due to elevated temperature varied across hybrids, ranging from 5.56% (DHM121) to 47.62% (DHM117). Similarly, the reduction in transpiration rate ranged from 2.80% (DHM121) to 36.76% (DHM117) under eT condition. Under eT + eCO_2_ condition, reduction in g_s_ ranged from 7.14% (DHM117) to 31.11% (900M GOLD) and for Tr it varied from 6.98% (DHM117) to 16.53% (900M GOLD). Notably, DHM117 had maximum recovery (> 90%) in g_s_ and Tr under eT + eCO_2_ condition, while a linear reduction was observed for 900M GOLD under both eT and eT + eCO_2_ conditions. DHM121 showed higher reduction in g_s_ and Tr under eT but displayed greater recovery under eT + eCO_2_ condition. In DHM117, the reduction in Tr due to eT was higher than the reduction in A_net,_ resulting in higher water use efficiency (WUE) compared to ambient condition. All hybrids recorded higher WUE under eT + eCO_2_ condition, where the presence of elevated CO_2_ helped in the recovery of A_net_ even with eT, leading to increased WUE (Fig. 3D). The impact of eCO_2_ was more prominent in reducing Tr of DHM121 and NK6240, while it improved A_net_ with DHM117 and 900M GOLD (Table 7).

**Figure 4:**
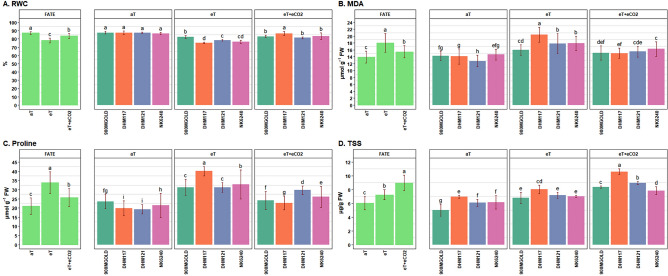
Performance of maize hybrids under different treatment conditions for physio-biochemical traits. Data are given as mean ± SD. Treatments with different grouping letters are significantly different.

To assess the leaf water status of plants, the relative water content (RWC) was estimated under different treatments (Fig. 4A). Under ambient condition, the RWC was approximately 87%, indicating good cellular hydration. However, eT caused a significant reduction in RWC, dropping to 78%. Interestingly, under the eT + eCO_2_, the RWC recovered to around 83%, demonstrating the positive effect of eCO_2_ in maintaining better leaf water status even under high temperature (Table 6). Among hybrids, 900M GOLD exhibited the ability to maintain a significantly higher RWC (82%) under eT compared to the other hybrids, while DHM117 was most affected (75%). However, DHM117 recovered substantially under eT + eCO_2_ (Table 7). Overall, the negative impact of eT was mitigated to some extent under eT + eCO_2_ treatment for all hybrids.

The level of lipid peroxidation, indicated by measurement of MDA content, exhibited a significant increase under the eT condition compared to ambient control (Table 6). Among hybrids, DHM117 showed the highest MDA content (20.47 mg/g fresh weight) under eT while 900M GOLD had the lowest level (15.99 mg/g fresh weight) (Fig. 4B). However, under eT + eCO_2_ condition the impact of eT was mitigated, resulting in lower MDA content.

There was significantly higher accumulation of proline in all the hybrids under eT condition (Fig. 4C). The accumulation of proline was more than double in DHM117 under eT as compared to ambient control. However, the impact eT was mitigated under eT + eCO_2,_ indicating the beneficial role of eCO_2_. Among the hybrids 900M GOLD was found to be more stable under different treatments in terms of proline accumulation (Table 7).

The hybrids had significantly lower free amino acid (FAA) compared to ambient condition (Table 6) under eT and eT + eCO_2_, although there was variation among hybrids. Among hybrids, 900M GOLD, DHM121 and NK6240 maintained similar FAA content under eT and eT + eCO_2_ conditions, while DHM117 showed reduced FAA accumulation. The eT and eT + eCO_2_ conditions led to increased TSS accumulation, with highest accumulation observed in DHM117. In contrast, 900M GOLD showed lowest TSS accumulation under eT, indicating that the metabolic activities of this hybrid were less affected under eT. The starch accumulation under different treatments did not vary much among hybrids under different treatments, although relatively higher starch (Fig. 4D) accumulation was observed under eT + eCO_2_ condition compared to ambient and eT (Table 7). Plants tend to remobilize more starch under elevated temperature and CO_2_ to provide energy and carbon when photosynthesis is potentially limited. The release of sugars and other derived metabolites support plant growth under stress and function as an osmo-protectants to mitigate the negative effect of stress. The carbon content did not show much variation among hybrids under different treatments, although relatively higher carbon accumulation was observed under eT + eCO_2_ condition compared to ambient and eT conditions.

## Discussion

A meta-analysis examining the impact of climate change on plants revealed clear evidence that physiological, growth, and yield related traits were influenced by eT and eCO_2_^60,61^. It is evident that crops respond to changing climatic conditions through intricate phenological, physiological, and biochemical processes. The primary objective of the present study was to assess the impact of elevated temperature individually and in combination with elevated CO_2_ (eT + eCO_2_) on maize, a C4 crop. Additionally, the study sought to quantify the role of elevated CO_2_ in mitigating the adverse impacts of elevated temperature on maize plants. By examining the interactive effects of these factors, the research aimed to provide an insight into the potential benefits of eCO_2_ in alleviating the ill effects of elevated temperature, specifically in the context of maize cultivation. The availability of diverse maize hybrids in the seed chain has also made it possible to estimate the variability among hybrids cultivated by farming communities under various production systems.

The intricate interplay between abiotic stresses, notably drought and elevated temperature, has been recognized for its discernible impact on the temporal dynamics of maize flowering, specifically influencing the tasselling, anthesis and silking in maize^62^. In our study, we investigated the ramifications of eT and the synergistic interplay of eT + eCO_2_ on phenology of flowering of maize. Specifically, eT accelerated the onset of anthesis and silking. This acceleration, however, was not replicated under the combined influence of elevated temperature and carbon dioxide (eT + eCO_2_), which intriguingly manifested behavior akin to ambient conditions. The ASI increased under elevated temperature (eT) conditions due to the early onset of anthesis compared to the requisite days for silk emergence. The variable responses exhibited by different hybrids under these conditions underscored their inherent genetic potential.

Among the hybrids, DHM117 exhibited a higher ASI under the set levels of elevated temperature condition, indicating its sensitivity to high temperature. In contrast, 900M GOLD displayed a relatively lower ASI, suggesting a greater tolerance or resilience to the set level of elevated temperature. The ASI is a critical trait in maize crop for ensuring successful fertilization and proper seed setting. These divergent responses of these hybrids to elevated temperature offer valuable insights into their adaptative capacities and performance under challenging environmental conditions. Aligning with existing research, our findings echo the trend of elevated temperature in reducing the days to anthesis or silking in maize hybrids^63^. In another study, it was observed that warmer temperature mainly affected the reproductive stages and thereby grain yield was significantly reduced to 80–90% as compared to normal condition^64^. In the present study, we made an interesting observation that the ASI of sensitive maize hybrids (DHM117 and DHM121) showed some degree of recovery under eT + CO_2_ condition. This finding highlights the potential ameliorative effect of elevated CO_2_ on phenological parameters. As climate change continues to exert its complex influence, the intricate relation between temperature, CO_2_, and maize crop invites for further exploration into the adaptive mechanisms that may shape agricultural resilience.

Generally, plants with different photosynthetic pathways exhibit a complex distinct response to eCO_2_ and temperature. The C3 crop species, known for their increased photosynthesis under eCO_2_ conditions, stand in stark contrast to the C4 plants, which, due to their efficient CO_2_ concentrating mechanism, exhibit a more modest enhancement in net photosynthesis and biomass. In the present study, A_net_ of maize hybrids was reduced at eT and, however the magnitude of response of individual hybrid varied. Among the hybrids, 900M GOLD recorded the highest per se values for A_net_ at all three conditions. Conversely, DHM117 exhibited a significant reduction in A_net_ under eT condition. however, it also demonstrated the ability to recover to ambient levels in the presence of eCO_2_. This observation becomes a key in understanding the mitigative potential of elevated CO_2_ on the negative impact of eT on maize, a C4 crop. The interplay between eT and eCO_2_ emerges as a crucial determinant, resulting in a smaller reduction in net photosynthetic rate as compared to eT alone. This suggests that elevated CO_2_ has an effective role in counteracting the adverse effects of elevated temperature on maize plants, emphasizing its potential as a protective factor in challenging climatic conditions. In contrast, another study reveals that maize demonstrates signs of CO_2_ saturation at ambient levels and displays a sluggish response to higher concentrations of CO_2_^65^. These findings indicate that elevated CO_2_ can effectively enhance the photosynthetic performance of maize, even under moderately increased temperature conditions. Further, studies revealed the occurrence of photosynthetic acclimation in maize plants following prolonged exposure to elevated levels of CO_2_^66^. While, in rice a C3 crop, reduced net photosynthetic rate by high temperature was mainly attributed to the reduction of chlorophyll content as well as activities of enzymes involved in photosynthesis^67^.

Delving further into stomatal conductance (g_s_) and transpiration rate (Tr), the response of different maize hybrids to elevated CO_2_ takes centre stage. The presence of elevated CO_2_ resulted in reduced g_s_ and Tr in 900M GOLD and DHM121, while a slight increase was observed in DHM117 and NK6240. The hybrid DHM121 displayed the lowest variation in g_s_ across the three conditions, indicating that the impact of eT and eCO_2_ on stomatal response is specific to the genotype. Plants employ diverse responses to adverse environmental conditions, including changes in stomatal function to cope with drought and heat stress^68,69^. Stomata open for CO_2_ absorption during photosynthesis but close to prevent water loss through transpiration^70^. Maize faces the challenge of managing both low water availability and high temperatures in rainfed ecology, leading to a dilemma of preventing water loss while addressing leaf heating. The paradox of water conservation and leaf cooling remains a critical question for maize cultivation under drought and high-temperature conditions.

In maize, the reduction in g_s_ and Tr under elevated CO_2_ conditions was consistent with previous findings^43^. The impact of eT on reduction in Tr was found to be significantly more pronounced than its effect on A_net_ resulting in an increase in WUE in DHM117, while a decrease in other maize hybrids. This divergence in responses among the hybrids underscored the complexity of their reactions to eT and eT + eCO_2_ conditions. Interestingly, the presence of eCO_2_, even at higher temperature demonstrated a compensatory effect by recovering A_net_ while simultaneously reducing Tr. Under eT + eCO_2_ condition, this dual action contributed to an overall increase in WUE than aT in all four hybrids. The study aligns with previous observations indicating that increased WUE under elevated CO_2_ can result from an increase in A_net_ or decrease in g_s_ or a combination of both^71^. In the specific context of the current investigation, the improved A_net_ and reduced Tr under eT + eCO_2_ condition emerged as the primary contributors to the higher WUE. This indicates the intricate interplay of environmental factors and plant physiological responses, emphasizing the need for a comprehensive approach when evaluating the impact of climate change variables on crop performance. The findings underscore the importance of considering multiple variables and their interactions to decipher the complexities associated with the optimization of WUE in maize.

Temperature and humidity play pivotal roles in shaping leaf photosynthetic rates, influencing key processes such as stomatal conductance (g_s_), and transpiration rate, as well as biochemical processes. Vapor pressure deficit (VPD), affects photosynthetic rates through its influence on leaf stomatal conductance. Stomatal closure mitigates excessive transpiration, preventing a corresponding decline in plant water potential. Importantly, evidence suggests that increasing VPD can inhibit photosynthesis which was also observed in the present study. A decline in transpiration rate (Tr) is typically noted in various crop species under elevated Vapor Pressure Deficit (VPD) conditions^72–74^. The reduction in Tr, resulting from the partial closure of stomata at high VPD, contributes to conservation of soil water. However, this leads to a simultaneous decline in CO_2_ assimilation due to the synchronization of water vapor and CO_2_ exchange by leaves and canopies^75^. Elevated levels of CO_2_, however, can offset the impact of abiotic stress on water status^76^. Furthermore, the rise in CO_2_ diminishes the sensitivity of assimilation rates, caused by high VPD and partial stomatal closure^77^.

The lipid peroxidation in terms of MDA content has also been used as a valuable stress indicator^78,79^. In our study, the MDA content of maize hybrids increased significantly under eT as compared to ambient condition. However, the presence of eCO_2_ mitigated this increase, maintaining MDA levels similar to ambient conditions. There was differential response among hybrids at eT indicating the differential genetic potential among hybrids to cope with climatic stresses. Notably, the hybrid 900M GOLD displayed similar levels of MDA across different treatments, suggesting its resilience and minimal susceptibility to environmental changes. Proline plays a critical role as an antioxidant, osmolyte and the stabilizer of cellular macromolecules and structural components of cell walls^80^. Elevated temperature induced higher proline accumulation across all hybrids, with 900M GOLD demonstrating comparatively lower proline accumulation, signalling its superior ability to manage cellular activities even under high-temperature conditions. The role of proline in imparting stress tolerance was also reported earlier in rice and maize^81,82^.

The soluble sugars are also critical in maintaining the cellular structure and growth of plants^83^. Soluble sugars help in maintaining the leaf water content and osmotic adjustment of plants that is affected by abiotic stresses^84,85^. Understanding the role of sugars under various abiotic stresses including drought and high temperature is pivotal in modulating several physiological processes^86^. Previous studies revealed that soluble sugars have a critical role as an osmo-protectant, regulating osmotic adjustment, providing membrane protection, and scavenging toxic reactive oxygen species under various types of stresses^87^. The starch is also a key molecule in mediating plant responses to abiotic stresses, such as water deficit, high salinity or extreme temperatures. Plants have a general mechanism of remobilizing starch to provide energy and carbon during periods when photosynthesis may be limited, especially under stressed conditions. The dynamics of soluble sugars and starch underpin a pivotal role in maintaining cellular structure and growth. The release of sugars and other derived metabolites serves to support plant growth under stress and acts as osmo-protectants to alleviate the negative effects of the stress^88^. However, the starch and soluble sugars content were not significantly affected in maize with moderate level of temperature rise and eCO_2_, but was significantly affected when the temperature was above the threshold level^89^. In our study, we observed higher accumulation of soluble sugars and starch content under eT and eT + eCO_2_ in comparison to ambient condition and there was also variation between the hybrids. This suggests that, as water was not a limiting factor, it might have contributed to increased accumulation of soluble sugars and starch, even under eT and eT + eCO_2_ condition, however it requires further investigation to revalidate these findings. The impact of elevated temperature on biomass and its allocation to vegetative and reproductive components was substantial. Particularly, under the eT condition, there was a significant reduction in stem biomass. However, eCO_2_ acts as a recuperative force, promoting biomass recovery even under elevated temperature for all four maize hybrids. Our observations align with the hypothesis of positive responses of C4 crops to eCO_2_, showcasing increased total biomass, further echoing the diverse responses documented in the literature^41,42^. The impact of elevated temperature (eT) on yield components was also significant, and the extent of this impact varied among different hybrids. Under eT condition, yield parameters such as cob weight, seed number and seed yield was reduced due to poor seed setting. Studies by Johnson^90^ and Stone^91^ also revealed that heat stress during pollen formation and pollen shedding is particularly detrimental leading poor seed set and ultimately poor seed yield. The temperature above 30 °C also damages the cell division and amyloplast replication of maize kernels, leading to reduce the grain size and ultimately poor yield^92^. In the present investigation, we observed differential responses of maize hybrids to eCO_2_ under eT. The positive impact of eCO_2_ on C4 crops, specially under eT was significant. The growth and yield components showed positive responses akin to those observed in C3 crops. The presence of eCO_2_ even under elevated temperature, mitigated the detrimental effects of eT, leading to positive responses in all aspects of phenological stages, physiological processes, biomass production, and overall yield parameters. The present study indicated that 900M GOLD was superior in performance among hybrids under elevated temperature and CO_2_ which can fetch better return to the farmers under these climatic stresses. Our study aligns with previous research and understanding of these dynamic relationship between plants and their environment, offering valuable cues for sustainable agricultural practices in the face of predicted climatic conditions.

## Conclusions

In conclusion, our study demonstrates that elevated temperature significantly affects various traits, spanning phenology, physiology, biochemistry, biomass, and grain yield. The responses of maize hybrids varied with these climatic stresses, where DHM117 and DHM121 exhibiting an increased ASI under eT as compared to NK6240 and 900M GOLD. However, the presence of elevated CO_2_ reduced ASI similar to ambient condition. The physiological parameters such as MDA and proline increased under elevated temperature but were partially alleviated by eCO_2_. Among hybrids, 900M GOLD demonstrated superior performance under eT and eT + eCO_2_ conditions, suggesting potential benefits of cultivating this hybrid for farmers facing climatic stresses. The study contributes valuable insights for sustainable agricultural practices in the context of evolving environmental challenges and breeding programs aimed at developing climate-ready maize hybrids.

## Data Availability

The raw datasets including weather data of crop seasons used and/or analysed during the current study available from the corresponding author on reasonable request.
